# Towards improvement in prediction of iodine value in edible oil system based on chemometric analysis of portable vibrational spectroscopic data

**DOI:** 10.1038/s41598-018-33022-9

**Published:** 2018-10-03

**Authors:** Hong Yan, Jixiong Zhang, Jingxian Gao, Yangming Huang, Yanmei Xiong, Shungeng Min

**Affiliations:** College of Science, China Agricultural University, Beijing, 100193 P.R. China

## Abstract

Iodine value (IV) is a significant parameter to illustrate the quality of edible oil. In this study, three portable spectroscopy devices were employed to determine IV in mixed edible oil system, a new Micro-Electro-Mechanical-System (MEMS) Fourier Transform Infrared Spectrometer (MEMS-FTIR), a MicroNIR^TM^1700 and an i-Raman Plus-785S. Quantitative model was built by Partial least squares (PLS) regression model and four variable selection methods were applied before PLS model, which are Monte Carlo uninformative variables elimination (MCUVE), competitive reweighted sampling (CARS), bootstrapping soft shrinkage approach (BOSS) and variable combination population analysis (VCPA). The coefficient of determination (R^2^), and the root mean square error prediction (RMSEP) were used as indicators for the predictability of the PLS models. In MicroNIR^TM^1700 dataset, MCUVE gave the lowest RMSEP (2.3440), in MEMS-FTIR dataset, CARS showed the best performance with RMSEP (2.2185), in i-Raman Plus-785S dataset, BOSS gave the lowest RMSEP (2.5058). They all had great improvements than full spectrum PLS model. Four variable selection methods take a smaller number of variables and perform significant superiority in prediction accuracy. It was demonstrated that three new portable instruments would be suitable for the on-site determination of edible oil quality in infrared and Raman field.

## Introduction

Edible oil has been widely used for making dishes such as salad or fried food. It can provide essential nutrients and energy. Some kinds of valuable edible oil are quite expensive, such as olive oil, sesame oil and perilla oil, which makes industries trying to adulterate with cheaper vegetable oils. The chemical and physical properties of edible oil are correlated to the properties of the corresponding feedstocks. For example, the degree of oil unsaturation, defined as the iodine value (IV), which is important to assess quality and grade of oil, and authentication test for both the consumers and food industries.

The official testing methods of IV are developed by the American Oil Chemists’ Society (AOCS) and the association of analytical communities (AOAC)^1^. However, the conventional method of titration in determining IV is ineffective since it uses highly toxic chemicals that are environmentally unfriendly, and the method is complex and time-consuming. Meanwhile, although GC and HPLC have been widely used for the quality control of edible oil, they still can’t meet the demand of detecting large sums of samples in short time.

Rapid, non-invasive and chemical free methods have been proposed for the determination of physical and chemical properties of edible oil, such as Fourier transform mid infrared (FT-MIR), Fourier transform near infrared (FT-NIR) and Raman (RS) spectroscopy^2,3^. The total degree of unsaturation was evaluated from the quantitative measurement of the v(C=C) band intensity and its relation to the intensity of the band related to the (CH_2_) scissoring. Chemometrics approaches supported all these methods, Partial least squares-discriminant analysis (PLS-DA), Decision tree, Random Forest and Artificial neural network (ANN). etc are commonly used methods for classification. Moreover, for multivariate regression calibration, as we know, partial least squares (PLS) is the most popular method until now.

The theory of FT-NIR is relied on the absorption of electromagnetic radiation which wavenumbers range from 800 to 2500 nm. The spectra produced by FT-NIR mainly corresponds to overtones and combinations of vibrational modes referring to C-H, C=C, C=O and N-H chemical bonds which arises from overlapping absorptions^2,4,5^. And it has proved to be a dependable tool for measurement of biological and chemical systems on account of wide range overtone bands. The main limitation of FT-NIR is its dependence on reference methods, its low sensitivity to minor constituents and its dependency on intricate calibration procedures^6,7^. So aiming to predict indirectly, appropriate chemometric tools should be used for multivariate calibration which are highly indispensable for the advanced technology of spectroscopy. Some computational approaches such as PCA and PLS, which allow the processing of abundant variables that then need data reduction process.

Raman spectroscopic technique is also based on the vibrational transitions occurring. Raman scattering depends on the change of the molecular polarizability and is useful for the *in vivo* or on-site study^8,9^. It is also widely used to analyze food components such as proteins, lipids, and water in food science.

The application of vibratory spectroscopy and chemometrics in oil has been reported by many researchers. Lucyna Dymińska *et al*.^10^ used infrared and Raman methods to determine the iodine values of unsaturated plant oil. Cleiton A. Nunes^11^ assessed quality parameters, adulteration and authenticity of edible oils and fats by vibrational method and chemometrics. Nor Fazila Rasaruddin *et al*.^12^ also tested the IV of palm oils by FT-NIR. Li *et al*.^13,14^ reported the use of FT-NIR for rapid measurement of iodine value, saponification number and cis and trans content of edible oil. However, fewer investigations about portable vibrational spectroscopy methods application were reported and influence of variable selection on Raman spectroscopy was rarely systematic studied. In our study, both BOSS and VCPA were first applied in Raman spectra.

Variable selection methods are well recognized in chemometrics and industrial applications. The elimination of variables which do not contribute to any inference is highly desirable for several reasons. For example, in NIR, absorption bands of fundamental frequency vibrations and combination of vibrations make it possible for quantitative analysis. Generally, NIR doesn’t need sample preparation. Several properties can be predicted according to a single spectrum simultaneously. However, adverse issues are also inevitable, as absorption bands are usually overlapping. Moreover, spectroscopy characterizes a chemical sample with thousands of wavelength variables, which may include lots of irrelevant information for calibrations like noise or background, often resulting in a negative effect to the whole modeling. Therefore, suitable chemometrics algorithm is necessary to deal with NIR spectrum, with the purpose to eliminate the uninformative variables effectively by using variable selections.

Vibrational spectroscopy has been proved to be a reliable method of rapidly determining the physical and chemical properties in edible oil. It has provided a responsive alternative for the commonly used methods applied in the industries. However, more applications of on-site test should be developed. This study has three proposes. First is to investigate the feasibility of using MEMS-FTIR, MicroNIR^TM^1700 and i-Raman Plus-785S to quantify IV of edible oil based on PLS regression models. Second is to investigate the influence of variable selection methods especially BOSS and VCPA on the robustness and predictability of calibration models developed by PLS. Last one is to demonstrate the potential of three portable devices for the on-site analysis of edible oils in the view of IV.

## Materials and Methods

### Agents and reagents

Potassiumiodide (AR, Sinopharm Chemical Reagent Co., Led. China), Sodium thiosulfate pentahydrate (AR, Sinopharm Chemical Reagent Co., Led. China), Cyclohexane (CP, Sinopharm Chemical Reagent Co., Led. China), glacial acetic acid (AR, Sinopharm Chemical Reagent Co., Led. China).

### Sample preparation

Soybean oil, olive oil, peanut oil and blend oil products were obtained from local supermarket. Iodine value were operated by the standard titration method which is based on the official methods introduced in the method for animal and vegetable fats and oils-determination of iodine value (ISO 3961:1996, MOD). 59 samples were prepared by mixing the four kinds of oil with the concentration of soybean oil, olive oil, peanut oil, blend oil from 0% to 85.46%, 0% to 69.34%, 0% to 88.35%, 0% to 85.46%, respectively.

### Instruments

#### MicroNIR1700

MicroNIR1700 is a micro NIR spectrometer developed and manufactured by JDSU. The instrument uses a Linear Variable Filter (LVF) as a light-splitting element. The LVF is a special band-pass filter, which is specially fabricated into a wedge-shaped coating in a specific direction. Since the center wavelength of the passband and the film Layer thickness, the wavelength of the filter penetrates linearly in the wedge direction, which plays a role of spectroscopy. LVF is coupled to a linear array detector (128-pixel uncooled InGaAs photodiode array). Dual integrated vacuum tungsten light source, 16-bit A/D converter.

#### MEMS-FTIR

MEMS-FTIR is a long wavelength near infrared spectrometry machine developed by HAMAMATSU in Japan. The MEMS-FTIR is a Fourier transform infrared spectrometer which is compact and with low cost. A Michelson interferometer and an infrared detector are grouped together in a small space. The MEMS-FTIR is formed by a fingertip size FT-IR engine, a control board, a photo-detector, input/output fibers, etc. Its size is 75 × 100 × 27 mm. Spectral measurement or absorption measurement can be done simply by connecting to a PC via USB. It is very suitable for on-set *in-situ* test analysis.

#### i-Raman Plus-785S

i-Raman Plus is a portable raman instrument developed by B&W Tek, Inc Company. It uses innovative intelligent spectral processing technology, high efficiency thin back-illuminated CCD detector, lower cooling temperature, resulting in better signal to noise ratio and higher dynamic range. The i-Raman® Plus-785S has a maximum integration time of up to 30 minutes and has the unique advantage of detecting weak Raman signals. It combines both high resolution and wide spectral range with spectral ranges up to 3200 cm^−1^ and optimal resolutions up to 4.5 cm^−1^.

### Spectral Data Acquisition

#### MicroNIR^TM^1700

Measurement wavelength range was 900–1700 nm, the resolution was 6–10 nm, integration time was 8 ms, background and dark current were calibrated every 30 minutes.

#### MEMS-FTIR

NIR spectra were collected with 5 mm quartz cuvette. The spectra were acquired over the range 1100~2100 nm (middle gain resolution, 2000 ms scans) at room temperature. Between each spectrum, the quartz cuvette was rinsed by the next sample.

#### i Raman Plus-785S

Raman spectra were acquired with 5 mm quartz cuvette over the range 175~3200 cm^−1^ at room temperature. The resolution is 4.5 cm^−1^. Dark current was calibrated every 30 minutes and background was collected by the next sample.

### Software

All the program codes and datasets computations were edited and applied in Matlab (V2016a, Mathworks, USA) with my computer (SSD) with the configuration Intel Core i5-4210U 2.4 GHz CPU, 8 GB RAM for analysis. The codes of CARS^15^, BOSS^16^, VCPA^17^ can be downloaded from the link of references, others are in-house codes.

## Theory

### MCUVE

UVE-PLS is developed based upon the analysis of the regression coefficient vector^18^. The stability criterion c is defined by

1 $${c}_{j}={\beta }_{j}/s({\beta }_{j}),\,j=1,\,2,\,\mathrm{.....}\,p$$

2 $$s({\beta }_{j})={(\sum _{i=1}^{n}\frac{{({\beta }_{ij}-{\beta }_{j})}^{2}}{n-1})}^{1/2}$$

Where ${c}_{j}$ is utilized on the conjunction of the addition of the original data and random variables, *β*_*j*_ is on half of the regression coefficients of the *j*th variable when ignore the *i*th calibration sample, and n is the calibration samples number. *β*_*j*_ denote the mean value, and *s*(*β*_*j*_) stands for the standard deviation of all *β*_*ij*_ for the *j*th variable, and *β*_*ij*_ is obtained through leave-one-out approach.

The criterion of eliminating redundant variables is achieved as the equation below:

3 $$|({c}_{j})| < |\max ({c}_{artif})|$$

Here $({c}_{j})$ stands for stability criterion of the *j*th variable in the dataset originally; and $|\max ({c}_{artif})|$ stands for the absolute value of the maximum value for $({c}_{j})$ from the added random variables.

In MCUVE, Monte Carlo sampling strategy is brought in the UVE instead of leave-one-out method: random choosing M samples from all the calibration samples to set up PLS models to calculate the regression coefficient *β*, then repeating the process for N times. So Eq. (2) convert into the following equation:

4 $$s({\beta }_{j})={(\sum _{i=1}^{N}\frac{{({\beta }_{ij}-{\beta }_{j})}^{2}}{N-1})}^{1/2}$$

Here, *β*_*ij*_ denotes the regression coefficient of the *j*th wavelength in partial regression model, which is established by the *i*th M random chosen samples.

### CARS

CARS is proposed also based on absolute value of regression coefficients with the purpose evaluating the significance of variables^15^. Monte Carlo is employed for sampling. To carry out feature selection and leaving out variables with small absolute regression coefficients in compulsive way, the exponentially decreasing function (EDF) is adopted. Through an EDF run, the ratio of wavelengths retained is processing in the *i*th sampling run follows the equation:

5 $${r}_{i}=a{e}^{-ki}$$

where *a* and k are two constants. They can be computed as:

6 $$a={(p/2)}^{1/(N-1)}$$

7 $$k=\,\mathrm{ln}\,(p/2)/(N-1)$$

Adaptive reweighted sampling (ARS) is adopted to realize a competitive feature selection in the view of the regression coefficients. This step follows the principle ‘survival of the fittest’ which is the basic theory of Darwin’s Evolution Theory^19^.

In the end, cross validation is employed to select the subsets according to the lowest RMSECV.

### BOSS

BOSS (The bootstrapping soft shrinkage) was developed by Baichuan Deng^16^ in 2016. This method is supposed to select informative variables with the existence of colinearity^20^. The steps are listed here:

1. K subsets are generated by using BSS, all the variables are assigned with equal weights (w).
2. Build K PLS sub-models with all the subsets and pick out best models with the lowest RMSECV.
3. Add up all the normalized regression vector to acquire new weights for variables.
  8 $${w}_{i}=\sum _{k=1}^{K}{b}_{i,k}$$
4. new subsets are generated by WBS according to new weights. This way guarantees that we have larger probabilities to select the variables which have larger absolute value of regression coefficients.

The subset which has the lowest RMSECV during the iteration is selected as the optimal variable subset by repeating step (2–4).

### VCPA

The optimizing variable subset is selected rely on binary matrix sampling (BMS) and EDF. In each iteration run, BMS and model population analysis (MPA) are carried out for once. After N EDF runs, 14 variables are remained which are considered be the most significant. Then RMSECV of all the combinations is calculated and the lowest RMSECV is recorded. In the end, the optimal subset with the lowest RMSECV is selected in the final run^17^.

### Partial Least Squares Regression (PLS)

PLS is a two-block regression method which is aimed to model the relationship between measured spectrum matrix X and a response vector y. Eqs (9) and (10) illustrate the PLS model^21^.

9 $$X=T{P}^{T}+{E}_{A}$$

10 $$y=T{q}^{T}+{f}_{A}$$

Here T is score matrix, P is the loading matrix. q as a y-loading vector, E_A_ and f_A_ are residual matrix of X and y-vector.

### Model Validation

To assess the performance of four promising variable selection approaches, namely CARS, SCARS, BOSS and SBOSS. Mean-centered was applied before modeling, and the optimal number of latent variables was determined by 5-fold cross validation. RMSEC (Root mean square error of calibration), RMSEP, ${{\rm{Q}}}_{{\rm{cv}}}^{2}$ and ${{\rm{Q}}}_{{\rm{test}}}^{2}$ were used to evaluate model performance. Standard deviation (SD) in 50 runs was employed to evaluate the robustness of PLS model. Simultaneously, the number of optimal latent variables (nLVs) and variables selected number (nVAR) were also reported.

11 $$RMSEC=\sqrt{{\sum }_{i=1}^{Ncal}{({y}_{i}-{\hat{y}}_{i})}^{2}/Ncal}$$

12 $${{Q}^{2}}_{cv}=1-{\sum }_{i=1}^{Ncal}{({y}_{i}-{\hat{y}}_{i})}^{2}/{\sum }_{i=1}^{Ncal}{({y}_{i}-{\bar{y}}_{i})}^{2}$$

While *y*_*i*_ is the experimental of the predicted properties, and ${\hat{y}}_{i}$ and ${\bar{y}}_{i}$ represent predicted and average respectively. *Ncal* is the number of calibration samples of the training set. RMSEP and ${{\rm{Q}}}_{{\rm{test}}}^{2}$ hold the equation following the same as RMSEC and ${{\rm{Q}}}_{{\rm{cv}}}^{2}$.

13 $$SD=\sqrt{\sum _{i=1}^{n}{({X}_{i}-\bar{X})}^{2}/(n-1)}$$

Each method was repeated for 50 times to assess the stability. The standard derivation (SD) was employed to calculate stability with Eq. (11). Where *X*_*i*_ and $\bar{X}$ are predicted and average value, separately. n stands for the number of all samples. The smaller the value of the stability, the more stable is the method.

## Results and Discussion

The dataset was separated into Calibration set (36 samples) and independent test set (23 samples) by K-Stone sampling^22,23^. For preprocessing, centering was employed in all datasets before modeling. For MCUVE, the Monte Carlo sampling number is set to 500. The regression coefficients of every variable were recorded. A coefficient matrix was developed after 500 iterations. Then, all the variables were ranked in accordance with their reliability index. In our study, 5-fold cross validation was employed to decide the number of variables. With all these settings, we ran MCUVE to estimate its predictive performance. For CARS run, the number of Monte Carlo sampling runs was 100. In BOSS, the bootstrap number was set to 1000. Several parameters also influence VCPA strategy, EDF runs (50 times), BMS sampling runs (1000), ω, the number of the left variables in the final run of EDF (14), σ, the ratio of best models of k sub-models (10%). We ran VCPA with the settings as in the parentheses. All the four variables selection methods were repeated for 50 times to assess the prediction accuracy and robustness.

### Infrared spectra features

**Figure 1:**
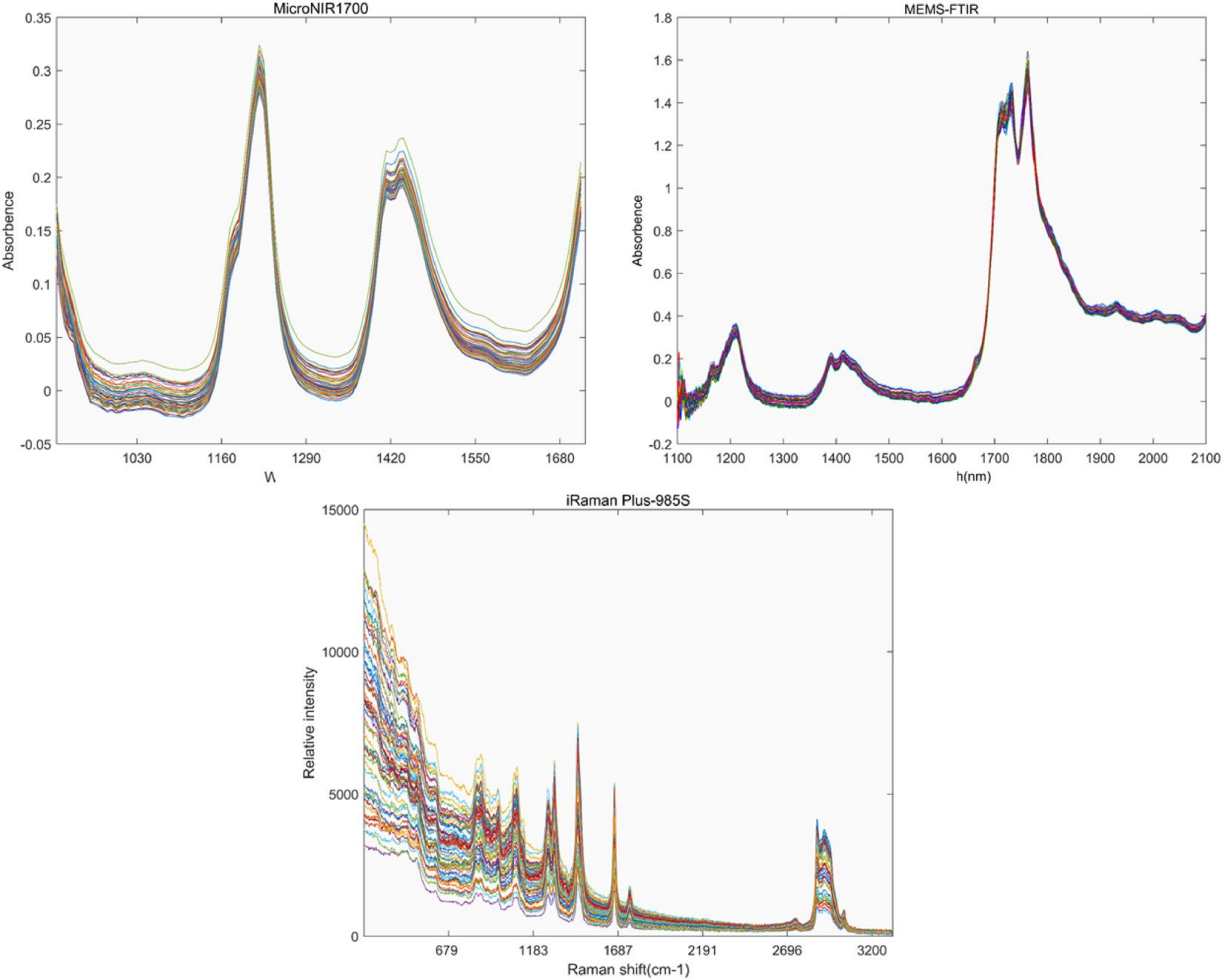
The raw spectrum of MicroNIR1700, MEMS-FTIR and iRaman Plus-985S.

Fig. 1 showed the raw spectra of mixed edible samples on MicroNIR1700, MEMS-FTIR and iRaman Plus-985S. MEMS-FTIR has wider spectrum range than.

MicroNIR1700 (1700~2100 nm). There are five absorption regions in MEMS-FTIR spectrum, which are in accordance with the studies that described the position of near infrared regions for edible oils. The two peaks which were centered around 1168 and 1210 nm linked to the second overtone of CH stretching vibration. The combination of the C-H stretching and vibration with other vibration modes of the concerned molecule associated with the regions around 1392 and 1414 nm. And two peaks centered 1726 nm and 1761 nm linked to the first overtone of the CH stretching vibration.

### Raman spectra features

**Figure 2:**
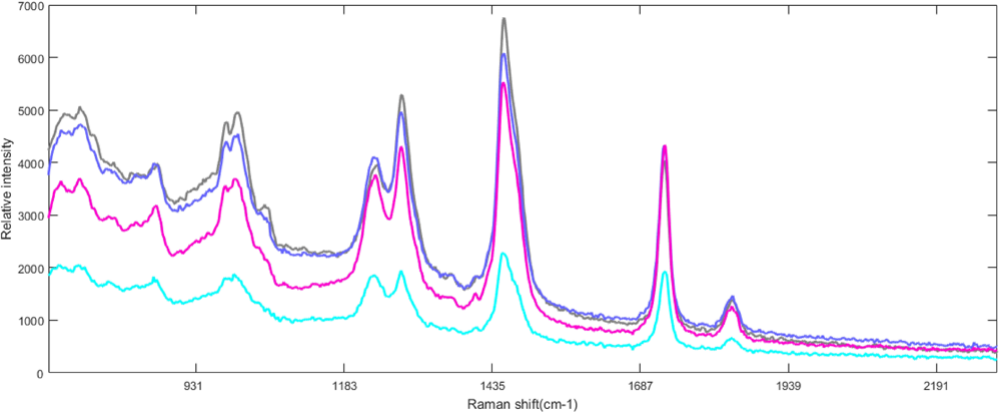
Selected raw Raman spectra (2190–678 cm−1) of mixed edible oil with different iodine values (IV = 86, 105, 113, 126) (Sample Number = 01, 20, 40, 58). The band assignment numbers correspond to the assignments provided in Table 1.

Four selected raw Raman spectra of mixed edible oil samples (IV = 86, 105, 113, 126) (Sample Number = 01, 20, 40, 58) were presented in Fig. 2. The Raman spectra assignment was provided in Table 1. The figure demonstrates that an increase at 1264, 970, 1296, 1128 and 1061 cm^−1^ as the IV increases.

**Table 1:** Assignment of the most common bands in Raman spectra of oil. The band numbers correspond to bands in Fig. 2.

Band number	Band position	Chemical group	Mode of vibration
1	1745	C=O	Stretch
2	1655	C=C	Stretch
3	1438	>CH_2_	Symmetric deformation (Scissor)
4	1301	>CH_2_	Twisting (All-in-phase)
5	1266	=C-H	Symmetric rock cis isomer
6	1125	C-C	Aliphatic in-phase stretch
7	1080	C-C	Aliphatic stretch
8	1068	C-C	Aliphatic out-of-phase stretch
9	970	=C-H	Out-of-plane bend cis isomer
10	868	C-C	Stretch

### Quantitation of IV by variable selection and PLS

**Figure 3:**
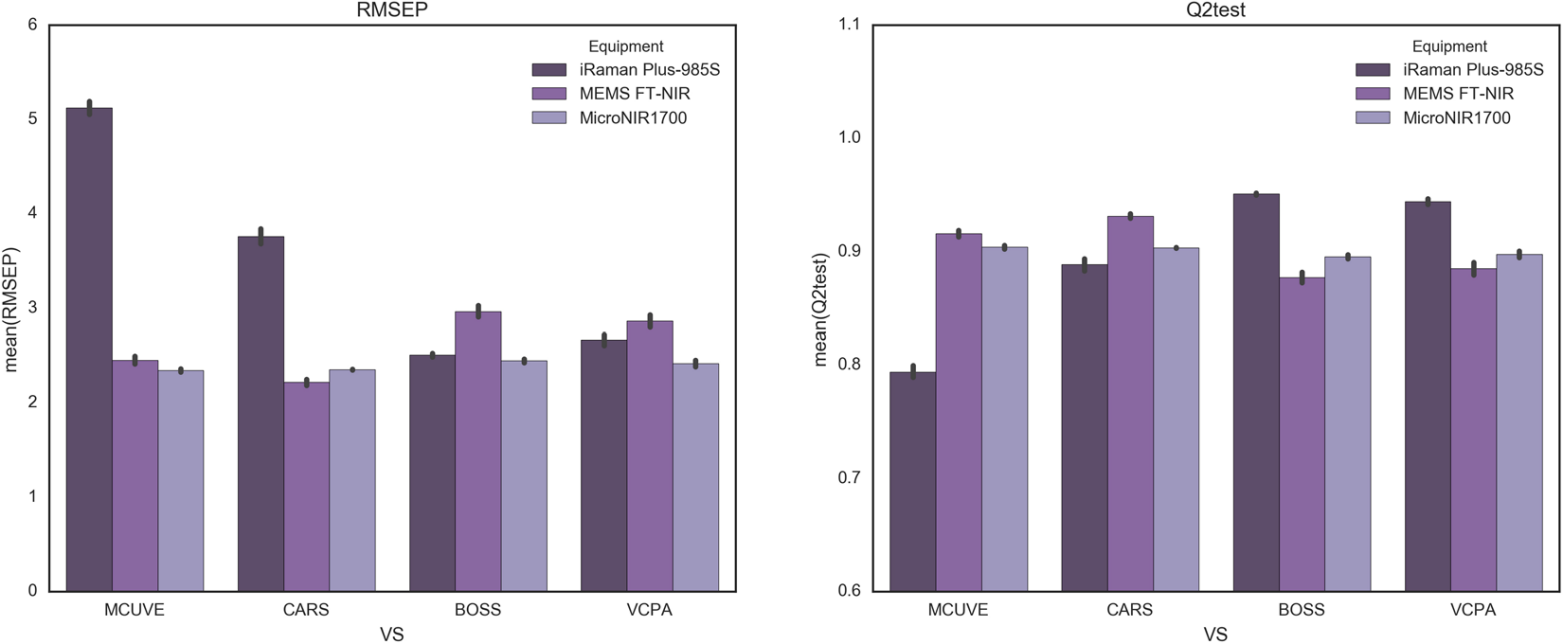
The Q2test and RMSEP of PLS, MCUVE, CARS, BOSS, and VCPA on MicroNIR1700, MEMS-FTIR and iRaman Plus-985S.

**Figure 4:**
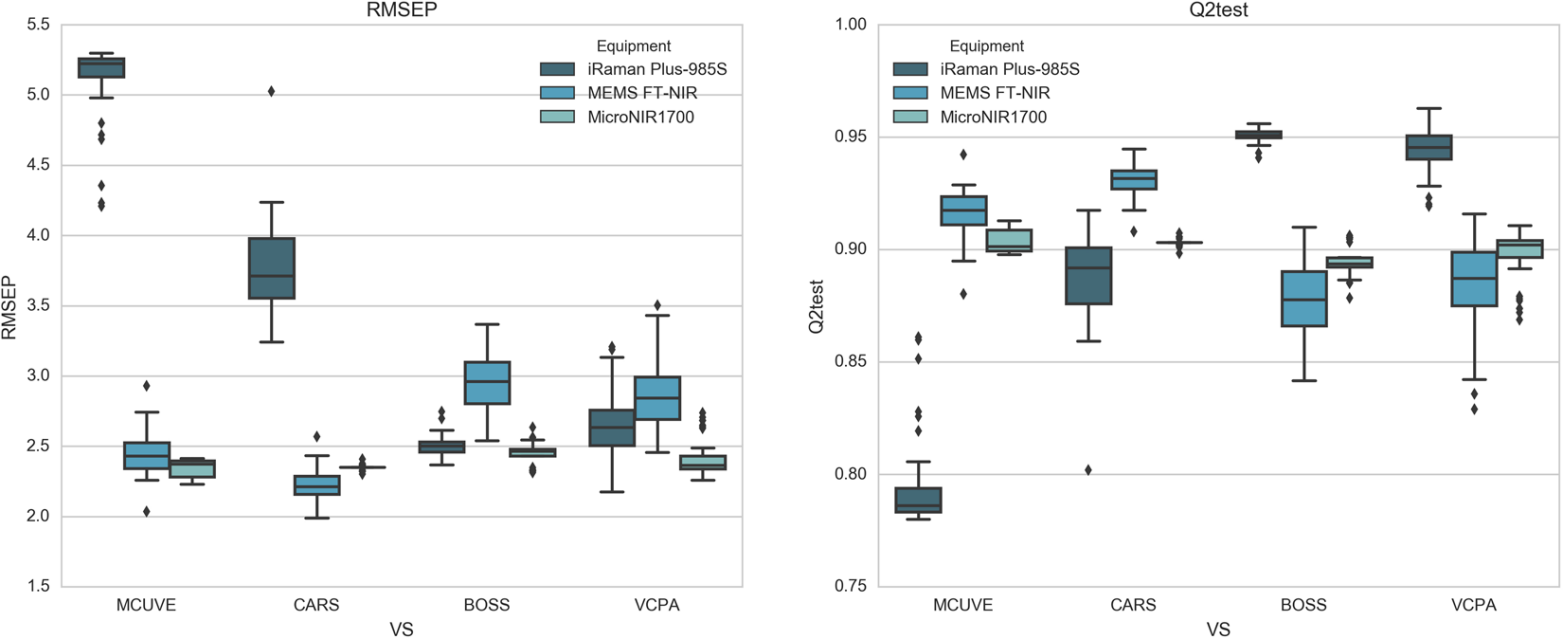
The boxplots of RMSEP and Q2test of 50 times run on MicroNIR1700, MEMS-FTIR and iRaman Plus-985S.

**Figure 5:**
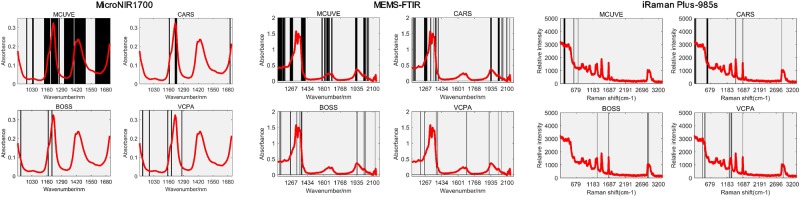
The variables selected by MCUVE, CARS, BOSS, and VCPA on MicroNIR1700, MEMS-FTIR and iRaman Plus-985S.

Table 2, Figs 3, 4 and 5 demonstrated the results of IV of edible oil. Both the mean and standard deviation were given in Table 2.

**Table 2:** The results on the MicroNIR1700, MEMS-FTIR and iRaman Plus-985S dataset of different variable selection methods.

Element	Characteristics	PLS	MCUVE		CARS		BOSS		VCPA	
MicroNIR1700			Results	SD	Results	SD	Results	SD	Results	SD
	nVAR	125	53	±28	4	±1	4	±1	7	±2
	nLV	3	3	±0	2	±0	2	±0	3	±0
	Q2_CV	0.9580	0.9643	±0.0016	0.9655	±0.0009	0.9693	±0.0009	0.9782	±0.0009
	Q2_test	0.9176	0.9038	±0.0051	0.9031	±0.0013	0.8953	±0.0064	0.8976	±0.0107
	RMSEC	2.6344	2.2008	±0.0474	2.1655	±0.0277	2.0412	±0.0285	1.7199	±0.0354
	RMSEP	2.3885	2.3440	±0.0628	2.3537	±0.0152	2.4455	±0.0756	2.4170	±0.1217
MEMS FT-NIR			Results	SD	Results	SD	Results	SD	Results	SD
	nVAR	3108	731.8	±557.808	216	±107	24	±6	11	±1
	nLV	3	2.36	±0.484873	3	±0	3	±0	4	±0
	Q2_CV	0.9781	0.9604	±0.0088	0.9724	±0.0077	0.9856	±0.0015	0.9899	±0.0011
	Q2_test	0.8993	0.9159	±0.0103	0.9313	±0.0071	0.8770	±0.0174	0.8849	±0.0194
	RMSEC	1.6999	2.1683	±0.2523	1.8038	±0.2413	1.3141	±0.0677	1.1019	±0.0582
	RMSEP	2.6271	2.4531	±0.1489	2.2185	±0.1144	2.9645	±0.2097	2.8654	±0.2359
iRaman Plus-985S			Results	SD	Results	SD	Results	SD	Results	SD
	nVAR	3122	2194	±856	54	±39	6	±1	9	±2
	nLV	3	3	±0	3	±0	2	±0	3	±0
	Q2_CV	0.8317	0.8044	±0.0136	0.8805	±0.0202	0.9524	±0.0023	0.9624	±0.0033
	Q2_test	0.8421	0.7940	±0.0194	0.8886	±0.0191	0.9508	±0.0028	0.9439	±0.0095
	RMSEC	4.6722	4.2588	±0.1583	3.3194	±0.2703	2.1009	±0.0499	1.8657	±0.0854
	RMSEP	3.9525	5.1221	±0.2575	3.7598	±0.3071	2.5058	±0.0703	2.6664	±0.2227

nVAR: The number of selected variables. nLVs: The number of selected latent variables of PLS.

RMSEC: Root mean square error of calibration. RMSEP: Root mean square error of prediction.

SD: Standard deviation in 50 runs.

As for MicroNIR1700, the four variable selection methods didn’t give great improvements compared to the full spectra PLS model. MCUVE gave the best performance with RMSEP (2.3440), it increased 1.86% than full spectrum PLS model. Compared to MCUVE, CARS, BOSS and VCPA choose much fewer variables (Fig. 5 and Table 2).

CARS achieved a good prediction with the least variables, we can see that from both Figs 4 and 5. the reason may be that variables are heavily collinear and therefore the model’s variance could be reduced with fewer variables. BOSS and VCPA also had fewer variables, but they retained variables around 700 nm and 800 nm which were uninformative, that was the reason why the model of BOSS and VCPA performed worse. There were no absorption peaks around 700 nm and 800 nm, so it didn’t have any information. Figure 2 (3) in Supplementary Materials showed the regression coefficient path of each variable from one run of CARS with the100 runs of sampling. We can see that in the first sampling run, the absolute value of regression coefficient of each variable was very small. However, with the number of sampling runs increased, the coefficients of some variables became larger and larger while others got smaller and smaller. Especially, the regression coefficients even decreased to zero if the relevant variables were knocked out by CARS. Therefore, the corresponding variable has more chances to survive if the absolute regression coefficient performs larger. It is also essential to analyze the regression coefficient path of each wavelength as shown in Fig. 2 (2)(3) of MEMS-FTIR dataset (Supplementary Materials). As previously mentioned, each line reflected the changing of regression coefficient of one variable. During CARS iteration, some significant variables were chosen while other ineligible ones were ignored.

In MEMS-FTIR dataset, CARS performed the best with the lowest RMSEP (2.2185), followed by MCUVE, VCPA and BOSS. CARS also got the minimum standard deviation (0.1144). The reason why CARS presented the best may be that it selected most of the informative variables around 1392 nm, 1414 nm which corresponded to the combination of the C-H stretching and vibration with other vibration modes of the concerned molecule. Most strong absorption bands of the calibration samples were observed at CH and CH_2_ over-tones. These overtones occurred at 1207, 1391, around 1408, 1715 and 1734 nm together with minor absorption bands in the range between 2083 and 2202 nm (Fig. 5).

The absorption bands around 1207 nm comprised the second overtones of C-H, while those between 1612–1818 nm were attributed to the first overtones of C-H which comprises CH_3_, CH_2_ and HC-CH. The reason why BOSS played the worst is that BOSS chose the variables with much noise (around 1100 and 2100 nm) and missed the informative variables (around 1715 nm).

As for Raman data, it is obvious to see that variable selection had a great influence to the PLS model. BOSS and VCPA improved a lot compared to the full spectrum PLS model, nevertheless MCUVE and CARS showed bad results. Figure 5 demonstrated selected variables of four variable selection methods. It is noticeable that both MCUVE and CARS retained the variables between 175 and 220 nm that are mainly noise. Variables belong to the region are uninformative, moreover, MCUVE missed the informative variables around 1745, 1655, 1438, 1301 and 1068 nm, hence MCUVE had a bad result even worse than full spectrum PLS model. It should be noticed that denoising ability of MCUVE and CARS are weak (Fig. 4). VCPA ranked the second place in all the models with good stability. It retained informative variables efficiently on account of the employment of BMS and MPA. BMS is a sampling approach that each variable has the same opportunity to take part in the sampling process, which let it be a suitable sampling choice of variable selection. Moreover, the same with CARS, EDF makes VCPA select fewer variables. The RMSECV of every EDF run was presented in Supplementary Material. It was demonstrated that the trend of decreased RMSECV is accordance with the EDF. The RMSECV is decreasing with the shrinking variable space, which means that the remaining variables were gradually selected toward the optimal variable subset. At last, the optimal subset from all the combinations among 14 variables was found.

In general, the results in Table 2 showed relatively good predictions which indicated that the calibration models are robust. It indicated that the predictions of MicroNIR1700, MEMS-FTIR and i-Raman-785s were comparable to their corresponding reference methods for IV determination and therefore the three portable devices based on edible oil analysis is suitable for on-site measurement of IV for edible oil or other biodiesel production. Variable selection is necessary for quantitative model to improve the prediction results and ensure the reliability.

## Conclusion

In our study, we discussed the influence that variable selection methods MCUVE, CARS, BOSS and VCPA has on the MicroNIR1700, MEMS-FTIR and i-Raman-785s PLS calibration modeling for the vibratory spectroscopy analysis IV of edible oil. The results showed that the three portable spectroscopy devices were capable of providing a rapid and accurate measurement of IV of edible oil destined for biodiesel production with a proper calibration and a responsive model. Once the calibrations are in place, portable device is a fast and easy to use method for the IV measurement in an on-site environment. It drastically reduces the time from routine IV value quality control analysis and does not involve the use of any chemical reagents. Conclusively, it’s possible to use portable vibratory spectroscopy as an edible oil quality control tool for IV measurement and more robust PLS and prediction models can be obtained based on variable selection methods.

## Electronic supplementary material

Supplementary material

## Data Availability

All data included in this study are available upon request by contact with the corresponding author.
