# Supplementary material for: Towards improvement in prediction of iodine value in edible oil system based on chemometric analysis of portable vibrational spectroscopic data
Source: Sci Rep. 2018 Oct 3;8:14729. doi: 10.1038/s41598-018-33022-9 (PMC6170483; doi:10.1038/s41598-018-33022-9)
Supplement: Supplementary file 1 — Supplementary material [file 41598_2018_33022_MOESM1_ESM.pdf]

Towards improvement in prediction of iodine value in edible oil system based on chemometric analysis of portable vibrational spectroscopic data

Hong Yan[a], Jixiong Zhang[a], Jingxian Gao[a], Yangming Huang[a],

Yanmei Xiong*[a], Shungeng Min**[a]

[a] College of Science, China Agricultural University, Beijing 100193, P.R. China

* Corresponding author.

** Corresponding author. Tel: +86 010 62733091; Fax: +86 010 62733091

Email addresses: xiongym@cau.edu.cn (Y. Xiong), minsg@cau.edu.cn (S. Min).

Fig.1. The changing of the number of retained variables and the average 5-fold RMSECV values of 50 times

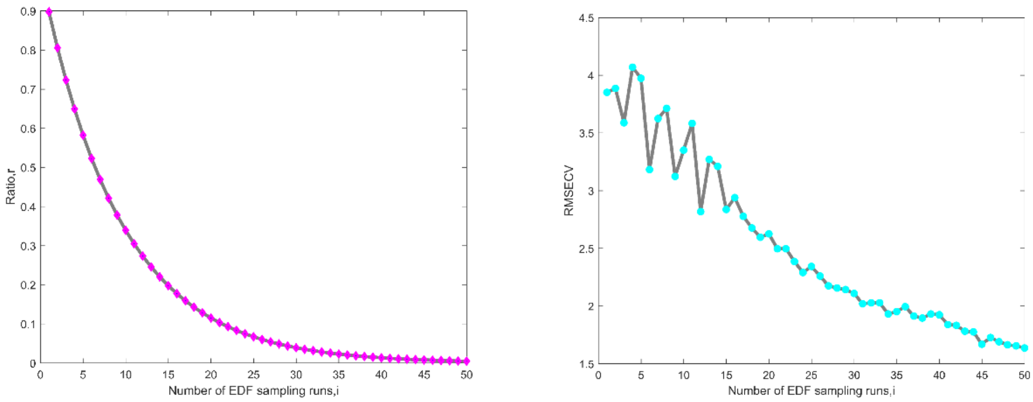

Fig.2. Plots (1)–(3), respectively, depict the changing of the number of sampled variables, 5-fold RMSECV values and the regression coefficient path of each variable. The vertical asterisk line denotes the optimal point where 5-fold RMSECV values achieve the lowest. MicroNIR1700, FTIR and iRaman Plus-985S.

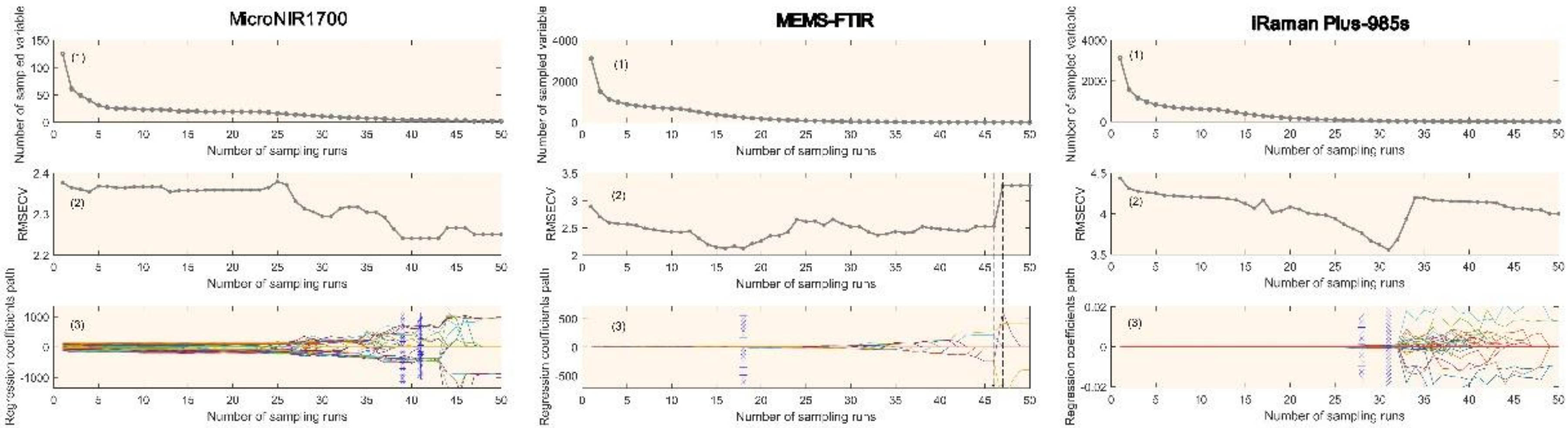

MicroNIR1700
MEMS-FTIR
IRaman Plus-985s
Number of sampled variable
Number of sampling runs
RMSECV
Regression coefficients path
(1)
(2)
(3)
